# Clinico-laboratory profile and perforin gene mutations of pediatric hemophagocytic lymphohistiocytosis cases: a five-year single center study

**DOI:** 10.11604/pamj.2020.36.354.25079

**Published:** 2020-08-27

**Authors:** Mohamed Abdelkader Almalky, Safaa Hamdy Ahmad Saleh, Eman Gamal Baz, Ahmed Elsadek Fakhr

**Affiliations:** 1 Pediatric Department, Faculty of Medicine, Zagazig University, Sharkia, Egypt,; 2 Microbiology and Immunology Department, Faculty of Medicine, Zagazig University, Sharkia, Egypt,; 3 Pathology Laboratory and Blood Bank, International Medical Center, Jeddah, Saudi Arabia

**Keywords:** Hemophagocytic lymphohistiocytosis, pediatric, diagnostic criteria, perforin, survival outcome

## Abstract

**Introduction:**

hemophagocytic lymphohistiocytosis (HLH) is an immunological disease characterized by hemophagocytosis of blood cells and proliferation of T-cells and histiocytes in the spleen and bone marrow then infiltration into body organs. Familial HLH (FHL) is a fatal disorder and determining gene mutations is a good guide for predicting the prognosis and choosing treatment options. This study aimed to illustrate the clinical, laboratory characteristics, including perforin gene mutation screening, treatment and survival outcome of pediatric HLH patients.

**Methods:**

we conducted this cross-sectional study on pediatric patients who were diagnosed with HLH using the revised HLH-2004 criteria, from January 2014 to February 2019 at Zagazig University Children's Hospital, Egypt. We collected demographic, clinical and laboratory data and screened for the presence of mutations in perforin (PRF1) gene by polymerase chain reaction (PCR) amplification. We treated the patients according to HLH-2004 treatment protocol and documented their survival outcome.

**Results:**

the total number of cases were 18; eight males and ten females, the age range was between three months and 12 years. Of the eight HLH-2004 diagnostic criteria, all patients met at least five criteria. We detected PRF1 gene mutation in 38.9% (7 patients) with nine previously unreported mutations. Sixteen patients (88.9%) received HLH-2004 treatment protocol and the remaining two patients died before initiation of treatment. The overall mortality was 72.2% (13 patients).

**Conclusion:**

our results increase the awareness of clinical and laboratory characterizations of pediatric HLH patients and the prevalence of PRF1 gene mutations among those patients.

## Introduction

HLH is an immunological disease characterized by hemophagocytosis of red bood cells (RBCs), platelets and neutrophils by histiocytes (activated macrophages) and proliferation of T-cells and histiocytes in the spleen, bone marrow and infiltration into body organs. The activated macrophages secrete large amounts of cytokines, which cause tissue damage and lead to organ failure [1]. In patients with HLH, a defect in the killing of the pathogens or cancer cells occurs due to an inherited defect in cytotoxicity in genetic forms of the disease, ending with inability to kill the target cell, hypercytokinemia and activation of macrophages. Serum cytokines such as IFN-γ, TNF-α, IL-6, IL-10 and IL-12 are highly elevated in addition to markers of immune activation such as soluble CD8, CD163 and CD25. Moreover, histopathological evaluation shows tissue infiltration by macrophages and lymphohistiocytes [2]. Classification of HLH includes two main groups; genetic (primary) and acquired (secondary). Molecular spectrum of genetic causes of HLH includes; FHL1 with unknown gene defect, FHL2 caused by PRF1 gene defect, FHL3 caused by UNC13D gene defect, FHL4 caused by STX11 gene defect, FHL5 caused by STXB2 gene defect, Chediak-Higashi syndrome caused by LYST gene defect, Hermansky-Pudlak caused by AP3B1 gene defect, Griscelli syndrome type 2 caused by RAB27A gene defect, X-linked proliferative type 1 caused by SHD2D1A gene defect and X-linked proliferative type 2 caused by BIRC4 gene defect [3].

For PRF1 gene, more than 120 different mutations have been detected: 101 missense/non-sense mutations and 21 deletion/insertion mutations [4]. The incidence of acquired forms of HLH is higher than the genetic forms. Secondary HLH has been originally described related to viral infections following organ transplantation and was previously described as virus associated HLH [5]. Later on bacterial, fungal and protozoal agents have been shown to be causative pathogens, as well. The well-known infectious organisms causing secondary HLH are Epstein-Barr Virus (EBV), cytomegalovirus (CMV), influenza viruses and Leishmania [6]. HLH may develop secondary to malignancies, metabolic disorders and autoimmune diseases [7]. Histiocyte society proposed HLH-2004 criteria as a guideline for diagnosis of patients with primary and secondary HLH. If the patient has a known genetic defect, the diagnosis of primary HLH is established. However, for patients without a known positive genetic defect, the diagnosis of either primary or secondary HLH can be done with the presence of at least five of the eight following diagnostic criteria: fever, splenomegaly, cytopenias ((at least bicytopenia), hemoglobin <9g/dL (neonates <10g/dl), platelet <100x10^9^/L, absolute neutrophil count <1x10^9^/L)), hypertriglyceridemia and/or hypofibrinogenemia (fasting serum triglyceride ≥3mmol/L (≥265mg/dl) plasma fibrinogen <1.5g/L), serum ferritin >500ng/ml, sCD25 (s IL-2 receptor) ≥2400U/ml, NK cell activity decreased (below 5%) or absent and hemophagocytosis (bone marrow, other tissues such as lymph nodes, cerebro-spinal fluid) [8]. The aim of this study was to illustrate the clinical and laboratory features, management, outcome and PRF1 gene mutation of HLH patients at Zagazig University Children´s Hospital.

## Methods

We conducted this cross-sectional study on pediatric patients diagnosed with HLH using the revised HLH-2004 criteria and they met at least five out of the eight diagnostic criteria [8], from January 2014 to February 2019, at Zagazig University Children´s Hospital in Egypt. Patients who met less than five of HLH-2004 diagnostic criteria were excluded from the study. We collected demographic, clinical and laboratory data of the patients, including: age, gender, presence of fever, hepatomegaly, splenomegaly, complete blood count, serum ferritin, triglycerides, fibrinogen, bone marrow aspirate, soluble CD25 level and NK cell activity. We treated the patients according to HLH-2004 treatment protocol [8] and documented their survival outcome. They received supportive care, including appropriate broad-spectrum antibiotics (until culture results are available), prophylactic cotrimoxazole, antimycotic therapy, antiviral therapy in patients with ongoing treatable viral infections, gastro-protection and intravenous immunoglobulin [8].

We screened our patients for the common viral infections triggering HLH including EBV, CMV and HBV using PCR techniques. Also, we searched for other causes of secondary HLH such as rheumatological diseases, malignancies, metabolic and autoimmune diseases in patients´ records. We screened patients for the presence of mutations in the coding exons and exon-intron boundaries of PRF1 gene by PCR amplification of genomic DNA followed by direct sequencing of the PCR products [9]. We performed the following steps: extracting genomic DNA from fresh peripheral blood using thermo scientific gene JET whole blood genomic DNA purification mini kit, amplification of the coding exons 2 and 3 of the PRF1 gene by PCR, using the following primers for exon2: 5' CCCTTCCATGTGCCCTGATAATC 3' and 5' AAGCAGCCTCCAAGTTTGATT 3'; and exon3: 5 'CCAGTCCTAGTTCTGCCCACTTAC 3' and 5' GAACCCCTTCAGTCCAAG CATAC 3', amplification of 500ng of DNA in a 50ul assay of 25ul dream taq green PCR master mix 0.4mmol/l of each primer and to the rest of volume water, reaction conditions were 3 minutes at 95°C followed by 30 cycles of 45 seconds at 95°C, 30 seconds at 60°C, 1 minute 45 seconds at 72°C; and then 10 minutes at 72°C, detection of the amplification product by agarose gel electrophoresis, primers used for sequencing were the same as those for amplification, cycle sequencing using the big dye terminator cycle sequencing reaction kit and separated the DNA fragments on an ABI prism 3700 DNA analyzer and finally comparing sequences with the published PRF1 gene sequence (Gen Bank accession no. M28393) using MEGA software (Molecular Evolutionary Genetic Analysis), basic local alignment search tool (BLAST) on The national center for biotechnology information (NCBI) and Chromas software.

Zagazig University institutional review board approved this study. Data were collected, extracted and analysed by SPSS version 20. Descriptive and analytical statistics were performed for all the studied variables. A number of statistical tests were used during the analysis process including Wilcoxon Mann-Whitney tests for comparisons of continuous data that were not normally distributed and X2 tests for comparisons of categorical data if all data were >5 (or Fisher´s exact tests if not).

## Results

This study included 18 patients who met at least five out of the eight HLH-2004 diagnostic criteria. Age of the studied patients ranged from 3 months to 12 years with a median 2.25 years. Eight patients were male and ten were female with 0.8: 1.0 male to female ratio. The values of laboratory characteristics of our patients are summarized in Table 1 and Table 2 shows the frequency and distribution of the studied patients according to clinical and laboratory data required in HLH diagnostic criteria. We detected PRF1 gene mutations which cause FHL2 in seven out of the 18 studied patients (38.9%) and interestingly, we found nine previously unreported mutations which have not been listed in the genomic databases on the Gene Bank using BLAST at NCBI. Table 3 shows molecular characterization of patients with PRF1 gene mutations. During screening our patients for the common viral infections and searching for secondary causes of HLH, we detected three patients with EBV, two patients with CMV and one patient with HBV. None of the patients with PRF1 gene mutations were positive for the screened viral infections. We didn´t detect any rheumatological diseases, malignancies, metabolic or autoimmune diseases. Sixteen patients (88.9%) received HLH-2004 treatment protocol and the remaining two patients died before initiation of treatment.

**Table 1:** laboratory data of the studied patients according to HLH diagnostic criteria

Laboratory data	Mean ± SD	Median	Range
ANC/mm^3^	1391.89 ± 962.23	930	400-3670
Hemoglobin level (g/dl)	8 ± 1.57	8	6-10
Platelet count (^*^10^3^/mm^3^)	87.72 ± 69.94	75.5	20-272
Triglycerides level (mg/dl)	280.78 ± 88.82	287	138-443
Fibrinogen level (mg/dl)	155 ± 80.28	141.5	45-340
sCD25 (U/L)	4478.72 ± 3457.22	16.5	660-11270
Ferritin level(ng/ml)	4481.94 ± 5043.89	3995	305-20000

**Table 2:** frequency and distribution of the studied patients according to clinical and laboratory data required in HLH diagnostic criteria

Clinical and laboratory data	N=18	%
Fever	18	100
**Splenomegaly**		
Yes	15	83.3
No	3	16.7
**Hepatomegaly**		
Yes	16	88.9
No	2	11.1
**Anemia (hemoglobin <9 g/dL)**		
Yes	10	55.6
No	8	44.4
**Thrombocytopenia (<100x10^9^/L)**		
Yes	16	88.9
No	2	11.1
**Hemophagocytosis:**		
Yes	8	44.4
No	10	55.6
**Elevated ferritin level (>500 ng/ml)**		
Yes	17	94.4
No	1	5.6
**Decreased fibrinogen level ( <1.5 g/L)**		
Yes	9	50
No	9	50
**Elevated triglycerides level (≥265 mg/dl)**		
Yes	10	55.6
No	8	44.4
**Decreased NK cell activity (<5%)**		
Yes	4	22.2
No	14	77.8
**Elevated sCD 25 (≥2400 U/ml)**		
Yes	11	61.1
No	7	38.9

**Table 3:** molecular characterization of patients with PRF1 gene mutations

Mutation type	Mutation subtype	Protein change	Site	Novelty	Outcome
**Frameshift mutation**					
P1	C 536 insertion	Framshift	Exon 3	Novel	Death
P2	C 536 insertion	Framshift	Exon 3	Novel	Death
P4	C 536 insertion	Framshift	Exon 3	Novel	Death
P5	C 536 insertion	Framshift	Exon 3	Novel	Death
P10	C 536 insertion	Framshift	Exon 3	Novel	Death
P14	C 536 insertion	Framshift	Exon 3	Novel	Death
**Silent point mutation**					
P4	900 C>T	No change	Exon 3	Known	Death
P10	519 A>G	No change	Exon 3	Novel	Death
P14	900 C>T	No change	Exon 3	Known	Death
**Missense point mutation**					
P2	1133 A>G	Asn>Ser	Exon 3	Novel	Death
P4	937 A>G	Asn>Asp	Exon 3	Novel	Death
P5	1259 T>C	Ile>Thr	Exon 3	Novel	Death
P10	443 T>C	Val>Ala	Exon 2	Novel	Death
P 13	422 T>G	Ile>Ser	Exon 2	Novel	Death
P 14	286 G>C	Gln>Arg	Exon 2	Novel	Death
	443 T>C	Val>Ala	Exon 2	Novel	
	626 G>A	Arg>Gln	Exon 3	Novel	
	937 A>G	Asn>Asp	Exon 3	Novel	

As regards patients' outcome, 13 patients died and five survived; two patients died before starting treatment, six patients died during induction period of HLH-2004 treatment protocol (before eight weeks of treatment) and five patients died during the maintenance period of treatment protocol (after eight weeks of treatment). According to patient survival; three patients with FHL2 survived after receiving successful hematopoietic stem cell transplantation (HSCT) and two patients with viral infections (one patient with EBV and the other with CMV) after completing HLH protocol and supportive treatment including antiviral therapy for CMV. As regards outcome in patients with PRF1 gene mutation; four out of the eight patients died during induction period of treatment (three patients did not find HLA matched donors and one patient was on the waiting list for HSCT) and the remaining three patients are alive on the last follow up after receiving HSCT. There was a significant negative correlation between patients´ age and their outcome (higher mortality occurred among younger). On the other hand, there was non-significant relation between patients´ outcome and gender, presence of fever, splenomegaly, hepatomegaly or laboratory data as shown in Table 4.

**Table 4:** correlation between patients' outcome and their demographic characteristics, clinical and laboratory data

Variables	Outcome		Test	
	Dead	Alive	Z/ χ2	p
	N=13(%)	N=5(%)		
**Age (year)**				
Mean SD	2.69 ± 2.88	6.1 ± 4.13	-1.729	0.049^*^
Median (range)	1.25(0.25-10)	6(2-12)		
**Gender**				
Male	6(46.2)	2(40)	Fisher	>0.999
Female	7(53.8)	3(60)		
**Fever**				
Yes	13(100)	5(100)	0	1
**Splenomegaly**				
Yes	10(76.9)	5(100)	Fisher	0.14
No	3(23.1)	0(0)		
**Hepatomegaly**				
Yes	13(100)	3(60)	Fisher	0.065
No	0(0)	2(40)		
**Bicytopenia**				
Yes	11(84.6)	5(100)	Fisher	>0.999
No	2(15.4)	0(0)		
**Decreased Fibrinogen level (<1.5 g/L)**				
Yes	6(46.2)	3(60)	Fisher	>0.999
No	7(53.8)	2(40)		
**Elevated Triglycerides (≥265 mg/dl)**				
Yes	8(61.5)	2(40)	Fisher	0.608
No	5(38.5)	3(60)		
**Decreased NK cell activity (<5%)**				
Yes	3(23.1)	1(20)	Fisher	>0.999
No	10(76.9)	4(80)		
**Elevated sCD25 (≥2400 U/ml)**				
Yes	8(61.5)	3(60)	Fisher	>0.999
No	5(38.5)	2(40)		
**Hemophagocytosis**				
Yes	7(53.8)	1(20)	Fisher	0.314
No	6(46.2)	4(80)		
**Elevated Ferritin level (>500 ng/ml)**				
Yes	13(100)	4(80)	Fisher	0.278
No	0(0)	1(20)		

Z: Mann Whitney test; ^*^p<0.05 is statistically significant

## Discussion

Nowadays, there is improved knowledge about the aetiology of HLH as a syndromic disorder with a unique pattern. However, persistent efforts are made to identify the genetic and the immunologic basis of this syndrome [10]. Our study showed that age of patients ranged from 3 months to 12 years with median age 2.25 years similar to Alzyoud *et al*. [11], Xu *et al*. [12], Sasan *et al*. [10] and Zhang *et al*. [13]. According to gender, male to female ratio was 0.8 similar to Tesi *et al*. [14] but differ from Alzyoud *et al*. Xu *et al*. and Sasan *et al*. who stated that male to female ratio of 1.2: 1, 1.27: 1 and 1.83: 1, respectively [10-12].

According to the diagnostic criteria of HLH, we found that all of our studied patients had fever and hyperferritinemia which were a common finding in many reports [11,15,16]. Fifteen patients (83.3%) and 16 patients (88.9%) had splenomegaly and hepatomegaly respectively, which was similar to Leow *et al*. [15] but differ from Alzyoud *et al*. [11] and Mukda *et al*. [16] whose studies reported that all patients with HLH had splenomegaly. Hepato-splenomegaly which is a result of direct organ infiltration by the activated immune cells is a cardinal sign of HLH [17] and is more frequently observed in pediatric patients [18]. About 89% of our patients had bicytopenia which was similar to Alzyoud *et al*. [11], Leow *et al*. [15] and Mukda *et al*. [16] but differ from Lee *et al*. [19] who reported that only 41% of patients had bicytopenia and according to their study, which was enrolled in the emergency department, only 25.8% of patients presented with the all required diagnostic of HLH upon admission, whereas most of the patients did not. Most of our patients had cytopenia which presence can be explained by two factors, the first is hemopoiesis suppression by the highly elevated levels of inflammatory cytokines, such as IFN-γ released by activated T-cells, and the second is the phagocytosis of blood cells by overactivated macrophages [20] despite hemophagocytosis was present in bone marrow of eight of our studied patients (44.4%) which was similar to Cetica *et al*. [21] but differ from most of other studies which denoted that most of the patients had hemophagocytosis [11,15,22-24]. Some clinicians may feel cautious to diagnose HLH in the lack of hemophagocytosis even though the fact that it may not be present early in the disease. In addition, clinicians may over interpret the finding of hemophagocytosis as a specific marker of HLH and this is not clearly evident [23]. Although bone marrow hemophagocytosis is fairly sensitive, it is not specific to establish a diagnosis of HLH [24]. Half of our patients had high triglyceride level (≥265mg/dl) and/or hypofibrinogenemia which were similar to the result reported by Lee *et al*. [19]. Meanwhile, Alzyoud *et al*. [11] and Leow *et al*. [15] had different finding concerning this aspect as they found that about 85% of their studied patient had this diagnostic criteria. Laboratory findings of HLH vary much between studies as regards age, time of diagnosis and whether it is primary or secondary HLH. Soluble CD25 was elevated above 2400U/ml in 11 patients (61%) which differ from Gregory J *et al*. [25] whose result was 42%. This difference can be explained by limited laboratory facilities in many institutions worldwide, like NK cell activity and soluble CD25 measurement. Four patients (22.2%) had decreased or absent NK activity which differ from Leow *et al*. [15] whose result was 57%. Assessment of NK cell activity can be done by different methods such as flowcytometry-based NK-cytotoxicity test (NK-cytotoxicity) and NK cell activity test for interferon-gamma (NKA-IFNγ) and this can explain different results obtained by different centres.

Concerning the cause of HLH, we detected seven patients with FHL2 and six patients with positive viral infection either for EBV, CMV or HBV. HLH in these patients could be either EBV-driven HLH, secondary HLH or genetic forms of FHL other than FHL2 triggered by infection as none of patients with PRF1 gene mutation in our study were positive for viral infection. Viral infection is a common triggering factor to primary or secondary HLH [26]. The rest five patients in our study needed further genetic studies and searching for other infectious agents to determine the cause of HLH.

In our study, direct sequencing of PCR products spanning the two coding exons (exon 2 and 3) of the PRF1 gene revealed mutations in seven out of 18 patients tested (38.9%), which is similar to Zhang *et al*. [13] and higher than the reported frequency of the Thailand population [16]. According to Zhang *et al*. [13], mutations in the PRF1 gene were predominant in Chinese children with primary HLH, similar to the results from Italian, Turkish and Korean groups [21,27,28]. This difference related to ethnic group variability. Several studies have reported different proportions of HLH genes across populations. In studies of Caucasian populations, the PRF1 gene has been reported as the most common causative genetic defect. PRF1 defective gene mutations (HLH2) were identified in approximately 60% of Caucasian HLH patients [9,29,30]. As regard patients´ outcome in our study, 13 patients (72.2%) died, two patients (11.1%) died before starting treatment, six patients (33.3%) died during induction period of HLH-2004 treatment protocol and five patients (27.7%) died during the maintenance period of HLH-2004 treatment protocol which was similar to the result reported by Sasan *et al*. [10] and differ from those reported by Elyamany *et al*. [31] and Kaya *et al*. [32] whose percentage of deaths were around 50%. Also, our results were on the contrary to Koh *et al*. Bin *et al*. Alzyoud *et al*. and Gregory *et al*. whose percentage of deaths were 31%, 26.7%, 19% and 21%, respectively [11,21,27,33].

This results has also been different from those of Bin *et al*. [32] whose study on HLH in Chinese children stated that HLH-related fatalities occurred as follows: (14.7%) of deaths occurred during the induction period of HLH-2004 treatment protocol weeks and (12%) of deaths occurred during maintenance period, also different from those of Koh *et al*. [27] whose result was as follows; 18% of deaths occurred before 8 weeks and 13% of deaths occurred after 8 weeks. Our study showed that higher mortality occurred among younger patients. This finding was similar to those of Koh *et al*. [27] and Kaya *et al*. [28] and differs from Gregory *et al*. [25] and Sasan *et al*. [10] who stated that there is no significant relation between patient age and outcome. On the other hand, there is a non-significant relation between our patients´ outcome and other clinical or laboratory criteria which might be due to the small sample size of patients investigated in our study. Bin *et al*. found that severe neutropenia was significantly associated with increased early death in HLH [33]. Our study limitations were the rarity of HLH disease, so picking up cases was an obstacle, delayed diagnosis of HLH due to confused clinical presentations with other disease and small sample size led to statistically non-significant results, although the presence of risk. So we recommend performing a multi-center study throughout extended period and larger sample size for more comprehensive data. We also recommend genetic studies to detect other types of familial HLH, other than PRF1 gene mutation which causes FHL2. The presence of bone marrow hemophagocytosis is fairly sensitive, so we recommend further studies to determine its specificity as a criterion in in HLH diagnosis.

## Conclusion

The majority of HLH patients had fever, hepato-splenomegaly, cytopenia and hyperferritinemia. Hemophagocytosis could not be detected in bone marrow of all patients and its sensitivity as an early diagnostic criterion should be revised. PRF1 gene mutation is a leading cause for familial HLH and we detected novel mutations related to it. HSCT is life saving in patients with FHL2. Our findings increase the awareness of the clinical and laboratory presentation and the prevalence of PRF1 gene mutations among paediatric patients with HLH.

### What is known about this topic

- The diagnosis of HLH can be done with the presence of at least five of the eight following diagnostic criteria: fever, splenomegaly, cytopenias, hypertriglyceridemia and/or hypofibrinogenemia hyperferritinemia, elevated sCD25, decreased or absent NK cell activity and hemophagocytosis;
- The PRF1 gene mutation causes FHL2.

### What this study adds

- Hemophagocytosis could not be detected in bone marrow of all patients and its sensitivity as an early diagnostic criterion should be revised;
- We detected novel mutations related to PRF1 gene.
